# Ambient insect pressure and recipient genotypes determine fecundity of transgenic crop‐weed rice hybrid progeny: Implications for environmental biosafety assessment

**DOI:** 10.1111/eva.12369

**Published:** 2016-03-02

**Authors:** Hui Xia, Hongbin Zhang, Wei Wang, Xiao Yang, Feng Wang, Jun Su, Hanbing Xia, Kai Xu, Xingxing Cai, Bao‐Rong Lu

**Affiliations:** ^1^ Ministry of Education Key Laboratory for Biodiversity and Ecological EngineeringFudan UniversityShanghaiChina; ^2^ Shanghai Agrobiological Gene CenterShanghaiChina; ^3^ Fujian Province Key Laboratory of Genetic Engineering for AgricultureFujian Academy of Agricultural SciencesFuzhouChina

**Keywords:** fitness, genetic background, hybrids, insect pressure, *Oryza sativa* f. *spontanea*, red rice, transgene introgression

## Abstract

Transgene introgression into crop weedy/wild relatives can provide natural selective advantages, probably causing undesirable environmental impact. The advantages are likely associated with factors such as transgenes, selective pressure, and genetic background of transgene recipients. To explore the role of the environment and background of transgene recipients in affecting the advantages, we estimated the fitness of crop‐weed hybrid lineages derived from crosses between marker‐free insect‐resistant transgenic (*Bt/CpTI*) rice with five weedy rice populations under varied insect pressure. Multiway anova indicated the significant effect of both transgenes and weedy rice genotypes on the performance of crop‐weed hybrid lineages in the high‐insect environment. Increased fecundity was detected in most transgene‐present F_1_ and F_2_ hybrid lineages under high‐insect pressure, but varied among crop‐weed hybrid lineages with different weedy rice parents. Increased fecundity of transgenic crop‐weed hybrid lineages was associated with the environmental insect pressure and genotypes of their weedy rice parents. The findings suggest that the fitness effects of an insect‐resistant transgene introgressed into weedy populations are not uniform across different environments and genotypes of the recipient plants that have acquired the transgene. Therefore, these factors should be considered when assessing the environmental impact of transgene flow to weedy or wild rice relatives.

## Introduction

The extensive cultivation and potential environmental release of genetic engineered (GE) crops have aroused worldwide debates over biosafety issues, including the potential environmental impact caused by transgene flow to weedy/wild relatives (Stewart et al. 2003; Andow and Zwahlen 2006; Ellstrand et al. 2013). It is argued that novel traits conveyed by transgenes with natural selection advantages, such as those resistant to biotic and abiotic stresses, may alter the fitness of populations of wild or weedy relatives that have picked up these transgenes through pollen‐mediated gene flow. Such transgene flow will possibly lead to undesirable ecological consequences (Stewart et al. 2003; Hails and Morley 2005; Lu and Snow 2005; Lu and Yang 2009; Warwick et al. 2009). Thus, the assessment of the potential ecological consequences caused by transgene flow to wild or weedy populations is required prior to the commercialization of a GE crop variety (Stewart et al. 2003; Lu and Yang 2009).

Given the difficulty of preventing transgene flow from a GE crop to its wild and weedy relatives, studying the fitness change of wild and weedy populations that have acquired transgenes is essential for assessing the potential environmental impact caused by transgene escape (Ellstrand et al. 1999; Ellstrand 2003; Song et al. 2003; Fenart et al. 2007). Many studies have been carried out to estimate the alteration of fitness and invasiveness by comparing GE crop‐wild or crop‐weed hybrid lineages with their wild or weedy parents (Arriola and Ellstrand 1997; Snow et al. 1998; Spencer and Snow 2001; Cao et al. 2009; Di et al. 2009; Huangfu et al. 2011). Conclusions from these studies vary. For example, Burke and Rieseberg (2003) examined the fitness effects of a transgene carrying resistance to the white mold disease in crop‐wild sunflower hybrids using a single accession of wild sunflower. They conclude that ‘the presence or absence of the *OxOx* transgene had no effect on seed output’ of the crop‐wild hybrids. Conversely, Snow et al. (2003) used 15 wild sunflower genotypes to test the fitness effect of crop‐wild hybrid lineages with or without a *Bt* insect‐resistant transgene (*cry1Ac*). Their results indicated that ‘reduced herbivore caused transgenic plants to produce an average of 55% more seeds per plant relative to nontransgenic controls’. Neither of the studies asked whether different recipient populations might have different responses. These contrasting results raise a question: Does the genetic background of transgene recipients or environmental conditions influence the fitness brought by a transgene?

Rice (*Oryza sativa*) is an important staple cereal crop that provides nutrition for nearly one‐half of the global population (FAO 2004). High‐yield and quality production of rice are essential for the world food security. The same species, weedy rice (*Oryza sativa* f. *spontanea*), is a noxious weed infesting rice fields all over the world (Delouche et al. 2007); it poses a great challenge for rice production. Studies from Asia, USA, and other countries showed that weedy rice infestations of cultivated rice can cause grain yield losses ranging from 5% to 100% (Delouche et al. 2007; He et al. 2014). Because of frequent natural introgression from cultivated and wild rice into weedy rice, weedy rice has accumulated tremendous genetic diversity (Cao et al. 2006; Xia et al. 2011a,b; He et al. 2014; Song et al. 2015), which poses a great challenge for weedy rice control.

The potential commercialization of GE rice varieties with different transgenes has stimulated worldwide concerns over environmental biosafety issues (Lu and Snow 2005; Lu and Yang 2009; Yang et al. 2011, 2012; Wang et al. 2014). One of these is the movement and spread of transgenes from GE rice to its coexisting weedy rice populations (Chen et al. 2004; Messeguer et al. 2004; Xia et al. 2011a). If transgenes with natural selective advantages are incorporated into weedy populations, weedy rice may become weedier or more invasive by enhanced fitness, as recorded in many other plants (Snow et al. 2003; Légère 2005; Warwick et al. 2008, 2009). In addition to the type of transgene, the environment where transgenes are exposed to and genetic backgrounds of transgene recipients should also be taken into consideration for the biosafety assessment.

To test the hypothesis that environment and recipients’ genetic background can affect the fitness of crop‐weed hybrids for assessing the potential environmental impact of transgene flow, we analyzed the field performance of fitness‐related traits in F_1_ and their F_2_ hybrid lineages derived from artificial crosses of an marker‐free insect‐resistant GE rice line (*Bt*/*CpTI*) and five weedy rice populations with different genotypes under varied insect pressure. The primary objective of this study is to address the impact of environmental insect pressure and genetic background of recipient weedy rice populations on the performance of fitness‐related traits in the transgene‐present F_1_ and F_2_ hybrid progeny. Findings of this study will add to our understanding of the fitness effects from transgene flow to diverse weedy populations under varied environmental conditions and consequently facilitate the assessment of its relevant environmental impact.

## Materials and methods

### Plant materials

Five weedy rice populations (W1, W2, W3, W4, and W5) with different origins, one insect‐resistant GE rice line (MF1), and its non‐GE rice parental line Minghui‐86 (MH86) were used to produce the crop‐weed F_1_ hybrids. The five weedy five populations with different agronomic traits (Table S1) were collected from Nepal, Vietnam, China, South Korea, and India, respectively. The insect‐resistant GE line contained one copy of the tightly linked *Bt/CpTI* transgenes engineered *via Agrobacterium*‐mediated transformation into a rice variety MH86 and bred through the T7 generation. The double‐T system (Komari et al. 1996) was applied to delete the selective marker gene (*hpt*, for hygromycin resistance). Theoretically, the only difference between the GE rice line and its non‐GE parent is the presence or absence of the transgenes. The *Bt* (*Cry1Ac*) gene was under the control of the maize ubiquitin (*Ubi*) promoter, and the *CpTI* (*cowpea trypsin inhibitor*) gene was under the control of the *ActID* promoter. The *Bt/CpTI* transgenes confer strong resistance to lepidopteron pests such as rice stem borers and rice leaf folders. The non‐GE parent MH86 is a widely used rice variety in China for rice production and hybrid rice breeding.

To compare crop‐weed hybrid lineages that contain transgenes (transgene‐present) with those that do not contain transgenes (transgene‐absent), F_1_ hybrids were produced through artificial crosses (hand‐pollination) between the five weedy rice populations and the GE or non‐GE rice lines. The GE and non‐GE rice lines were used as pollen donors, whereas the weedy rice populations were pollen recipients as would be the case under natural transgene flow. Consequently, ten combinations of crop‐weed F_1_ hybrids, either with or without the *Bt/CpTI* transgenes, were produced: five transgene‐present F_1_ hybrid lineages [coded as W1–W5‐F_1_ (+)] and five transgene‐absent hybrid lineages [W1–W5‐F_1_ (−)]. Five transgene‐present and transgene‐absent F_2_ hybrid lineages were also generated from each of the transgene‐present F_1_ hybrid combinations through self‐pollination. All F_2_ plants were examined for the presence or absence of the transgenes using the Colloidal Gold Strip for the *Bt* transgene (EnviroLogix Inc., Portland, ME, USA).

### Experimental design

Field experiments for characterizing the fitness‐related traits in F_1_ and F_2_ hybrid lineages were conducted in the designated Biosafety Assessment Centers of Fujian Academy of Agricultural Sciences, Fuzhou, China, in 2009 and 2011, respectively. Two types of environments (blocks) with the ‘high‐insect’ and ‘low‐insect’ pressure were established to estimate the fitness differences of the crop‐weed hybrid lineages. The high‐insect environment was achieved using UV light to attract insects and without insecticide spraying, whereas the low‐insect environment was achieved by spraying chemical insecticides (methamidophos, omethoate, buprofezin, or monosultap) once a week during the cultivation season to control insects (Yang et al. 2015).

Given that nearly no significant fitness cost was detected from the insect‐resistant transgenes in our pilot experiment under low‐insect pressure, the experiments for F_1_ and F_2_ hybrid progeny mainly focused on examining the fitness benefit from the insect‐resistant transgenes under the high‐insect conditions. The field experiments involved two planting designs: pure planting of each set of the plant materials in a plot; and mixed planting of equal number of transgene‐present and transgene‐absent plants alternately in a plot. Consequently, the experiments for F_1_ and F_2_ hybrid lineages each contained a total of ten pure‐planting treatments (transgene‐present [W1–W5‐F_1_/F_2_ (+)] versus transgene‐absent [W1–W5‐F_1_/F_2_ (−)] lineages), and five mixed‐planting treatments (mixture of equal number of transgene‐present and transgene‐absent plants) in the high‐insect environment (Tables 3 and Tables 4). In addition, five weedy rice populations (W1–W5) were used as controls in the F_2_ experiment (Table S1). Each treatment contained eight replicates.

Seeds of all the experimental materials were first soaked in water for two days and then germinated on a nursery bed. Seedlings were transplanted to experimental plots about one month after germination in a 6‐row × 6‐hill grid with 25 cm intervals between rows and hills. The experimental field layout was complete randomized blocking design. Field management followed the typical rice field management style of Fuzhou, Fujian province.

### Data collection and analysis

Fitness‐related traits were measured from eighteen plants randomly selected plants in each plot (Table S2). Leaves damaged by leaf‐rollers and tillers blasted by stem borers were recorded based on weedy rice plants and transgene‐absent hybrid plants, respectively, from pure and mixed‐planting plots, 60 days after seedling transplanting. An insect damage index (%) was calculated as the average percentage of the damaged leaves and blasted tillers (Xia et al. 2011b).

A three‐way anova was conducted to detect the influence of transgene presence versus absence, weedy rice genotype (per population source), and pure‐ versus mixed‐planting mode on the fitness‐related traits in the F_1_ and F_2_ hybrid lineages in the high‐insect environment. To determine differences in the fitness‐related traits, an independent *t‐*test was conducted between transgene‐present and transgene‐absent plants in the pure planting; and the paired *t*‐test was conducted between transgene‐present and transgene‐absent plants in the mixed planting. The Levene test was conducted to examine the homogeneity of variance. Insect damage index values were square arctangent logarithmic transformed for the test of homogeneity of variance.

The five weedy rice populations may have genetically different fecundity responses (susceptibilities) to the target insects. The fecundity response of a weedy rice population to the target insects was determined as the reduced percentage of seed production by every 1% increase in insect damage index in the high‐insect environment. Assuming that the fecundity response of crop‐weed hybrid lineages was inherited from their weedy rice progenitors, the fecundity increase from the transgenes in the transgene‐present hybrid lineages was simulated as: [insect damage index of transgene‐absent plants – insect damage index of transgene‐present plants] × fecundity response of weedy rice parents. The correlations between the observed fecundity increase in transgene‐present hybrid lineages with the specific insect damage index, the fecundity response of weedy rice to target insects, and the simulated fecundity increase were analyzed. The simulated fecundity increase was calculated as the fecundity response from every 1% insect damage index reduction in weedy rice parents multiplied by the insect damage index transgene‐absent F_1_ and F_2_ hybrid lineages. To study the effect of genotypes independently, correlation analysis was further conducted between the weighted fecundity increases (observed fecundity increase/insect damage index) and the fecundity responses to target insects of weedy rice populations. All statistical analyses were performed using the software SPSS ver. 15.0 for windows (SPSS Inc., Chicago, IL, USA, 2006).

## Results

### Insect pressure on weedy rice parents and transgene‐absent F_1_–F_2_ hybrid populations in the high‐insect environment

**Figure 1:**
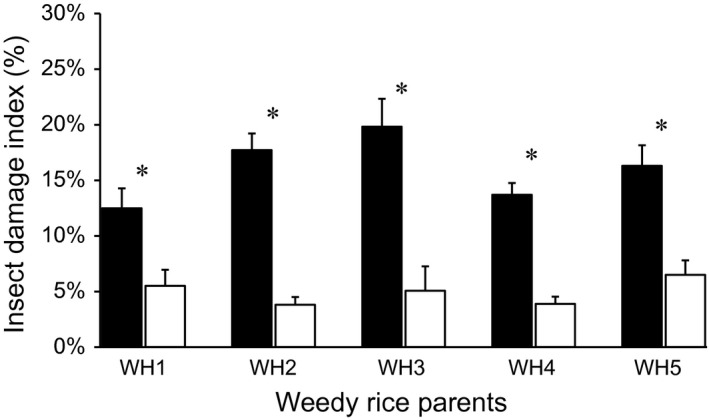
Insect damage index measured from weedy rice parents (W1–W5) in the high (black column) and low (white column) insect environment. Vertical bars: Standard errors (SE). *Significance at *P *<* *0.05.

**Figure 2:**
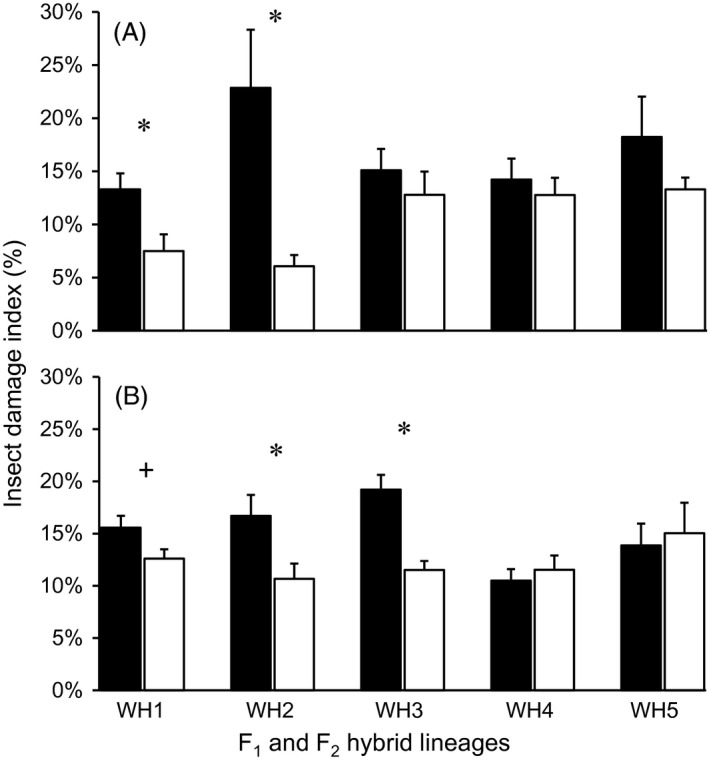
Insect damage index measured from transgene‐absent crop‐weed F_1_ (A) and F_2_ (B) hybrid lineages (WH1–WH5) in pure (black column) and mixed (white column) planting. Vertical bars: Standard errors (SE). +: Close to significance at *P *<* *0.1*: Significance at *P *<* *0.05.

A comparatively high level (13.5–19.8%) of insect damage index was detected in weedy rice parental populations in the high‐insect blocks (Fig. 1). The equivalent level of insect damage index was observed in F_1_ and F_2_ transgene‐absent lineages in the pure (10.5–22.9%) and mixed (6.1–15.0%) plantings (Fig. 2A,B). Notably, the insect damage index in transgene‐present F_1_ (0.39–2.90%) and F_2_ (3.12–4.60%) lineages was significantly lower than that in the corresponding transgene‐absent lineages based on the independent *t*‐test (Table S1). Moreover, the insect damage index in pure planting is significantly higher (*P* < 0.01) in pure planting (15.8 ± 1.2%) than in mixed planting (11.6 ± 0.9%). The considerably high levels of insect pressure in the high‐insect blocks as indicated by the insect damage index in the transgene‐absent lineages allowed us to estimate the fitness benefit of the crop‐weed hybrid lineages containing insect‐resistant transgenes.

Insect damage indexes varied considerably among the five weedy rice populations (Fig. 1), suggesting different responses of weedy parents to insect attacks. For example, a one‐way anova (LSD method) revealed the insect damage index in the W3‐population was significantly higher than that in the W1‐population (*P *<* *0.05) and W4‐population (*P *<* *0.05). Likewise, variation in insect damage index was also detected among the F_1_ and F_2_ transgene‐absent hybrid lineages derived from the five weedy parental populations, both in pure and mixed planting (Fig. 2A,B). Notably, the insect damage index recorded in the mixed planting of F_1_ and F_2_ hybrid lineages was generally lower than that in the pure planting (Fig. 2A,B), suggesting that mixed planting of transgene‐present and transgene‐absent plants reduced the ambient insect pressure.

### Fitness‐related traits in transgene‐present and transgene‐absent hybrid lineages

A three‐way anova indicated that the factors of transgene (transgenes versus nontransgenes) and genotype (weedy rice population source) had a significant effect on all the measured fitness‐related traits both in F_1_ and F_2_ hybrid lineages (Tables 1 and 2). These results suggested that both insect‐resistant transgenes and genetic ancestry from different weedy rice parental populations had a strong impact on fitness‐related traits of crop‐weed hybrid lineages. The factor of Planting Mode (pure versus mixed) also showed a significant effect on all the measured fitness‐related traits in F_1_ hybrid lineages (Table 1) and (except for one trait, number of panicles/plant) in F_2_ hybrid lineages (Table 2) indicating the complicated impact of mixed planting on the fitness. Except for the number of tillers per plant in the F_1_ hybrid lineage, the anova showed no interaction effect between the factors on the fitness‐related traits (Table 1).

**Table 1:** Three‐way anova for the fitness‐related traits of transgene‐present and transgene‐absent F_1_ hybrid lineages in pure and mixed planting. Three factors (transgene, genotype of weedy rice accessions, and planting mode) were included in the analysis. Transgene (T) had two levels (with or without transgenes). Genotype (G) had five levels (five weedy rice populations). Planting mode (C) had two levels (pure versus mixed planting)

Trait	Transgene (T)		Genotype (G)		Planting (C)		T * G		T * C		G * C		T * G * C	
	df	*F*	df	*F*	df	*F*	df	*F*	df	*F*	df	*F*	df	*F*
Number of tillers per plant	1	36.02***	4	18.39***	1	13.89***	5	2.71*	4	3.53	4	1.25	4	0.15
Number of panicles per plant	1	26.03***	4	20.88***	1	11.45**	5	1.73	4	0.78	4	1.38	4	0.71
Number of filled seeds per plant	1	46.03***	4	5.63***	1	10.51**	5	0.18	4	0.20	4	0.23	4	0.40
Ratio of seed set	1	25.14***	4	5.05***	1	5.69*	5	1.53	4	0.91	4	1.32	4	0.10
1000‐seed weight	1	16.14***	4	21.72***	1	4.47*	5	1.29	4	0.18	4	1.20	4	0.25

*F*,* F* value; *, **, or ***significant at *P *<* *0.05, *P *<* *0.01, or *P *<* *0.001.

**Table 2:** Three‐way anova for the fitness‐related traits of transgene‐present and transgene‐absent F_2_ hybrid lineages in pure and mixed planting. Three factors (transgene, genotype of weedy rice accessions, and planting mode) were included in the analysis. Transgene (T) had two levels (with or without transgenes). Genotype (G) had five levels (five weedy rice populations). Planting mode (C) had two levels (pure versus mixed planting)

Trait	Transgene (T)		Genotype (G)		Planting (C)		T * B		T * C		G * C		T * G * C	
	df	*F*	df	*F*	df	*F*	df	*F*	df	*F*	df	*F*	df	*F*
Number of tillers per plant	1	4.4*	4	19.1**	1	0.1	4	1.2	1	1.2	4	1.2	4	2.0
Number of panicles per plant	1	27.3**	4	17.3**	1	28.8**	4	0.4	1	0.1	4	1.8	4	1.0
Number of filled seeds per plant	1	112.5**	4	6.1**	1	0.2	4	1.5	1	0.0	4	1.3	4	0.9
Ratio of seed set	1	14.8**	4	3.8*	1	1.9	4	1.3	1	0.3	4	1.3	4	1.3
1000‐seed weight	1	8.3*	4	12.5**	1	1.4	4	1.2	1	0.1	4	2.2	4	0.2

*F*,* F* value; * or **significant at *P *<* *0.01 or *P *<* *0.001.

**Figure 3:**
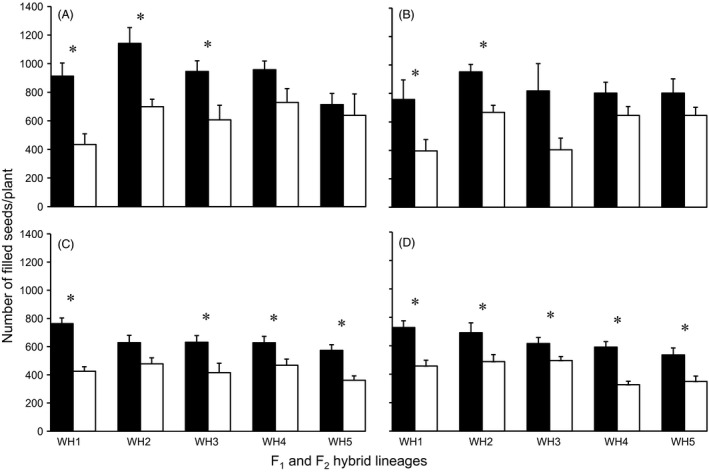
Number of filled seeds per plant in F_1_ (A, B) and F_2_ (C, D) hybrid lineages in pure (A, C) or mixed (B, D) planting in high‐insect environment. Black columns: Transgene‐present plants. White columns: Transgene‐absent plants. Vertical bars: Standard errors (SE). *: Significance at *P *<* *0.05. WH1–WH5: Crop‐weed hybrid lineages with different weedy rice parents.

Fitness‐related differences associated with the presence of insect‐resistant transgenes were estimated by comparing performance of the fitness‐related traits between transgene‐present and transgene‐absent hybrid lineages. Under high‐insect pressure, transgene‐present plants produced significantly more filled seeds than transgene‐absent F_1_ hybrids (Fig. 3A) in pure planting, although with great variation; transgene‐present F_1_ hybrid plants [W1–W5‐F_1_ (+)] produced 109.9%, 63.4%, 55.8%, 31.4%, and 11.7% more seeds than corresponding transgene‐absent hybrids. Similar fitness differences were observed for the transgene‐present F_2_ hybrid lineages [W1–W5‐F_2_ (+)] which produced 79.5%, 31.3%, 51.9%, 34.0%, and 58.7% more seeds than the comparable transgene‐absent hybrids (Fig. 3C) in pure planting. Other fitness‐related traits such as number of panicles and ratio of seed set showed significantly greater values in some of the transgene‐present hybrid lineages (Table 3) of low background resistance (W1‐F_1_ and W1‐F_2_) or with relatively higher insect damage index (W2‐F_1_). Under mixed planting, transgene‐present F_1_ and F_2_ hybrid lineages also produced significantly more filled seeds than the transgene‐absent counterparts (Fig. 3B,D), although also with great variation. Due to the relative lower insect damage index in the mixed planting, only few other fitness‐related traits were detected significant differences between transgene‐present and transgene‐absent hybrid lineages. However, the transgene‐present hybrids produced significantly more tillers than the transgene‐absent hybrids in mixed planting (Table 4), suggesting a stronger competitive ability of transgene‐present plants than transgene‐absent plants in the F_1_ hybrid population.

**Table 3:** Independent *t*‐tests for fitness‐related traits between transgene‐present (+) and transgene‐absent (−) hybrid lineages in pure planting. Values in brackets indicate standard errors (SE). Values in **bold** followed by asterisks indicate significant differences between the corresponding transgene‐present and transgene‐absent lineages

Code of weed rice and hybrids	No. of tillers per plant	No. of panicles per plant	Ratio of seed set (%)	1000‐seed weight (g)
WH1‐F_1_ (+)	18.6 (1.4)	14.4 (1.1)	**42.3 (2.8)** a	**22.8 (0.4)** a
WH1‐F_1_ (−)	16.4 (1.5)	13.2 (1.0)	30.6 (3.1)	21.5 (0.4)
WH2‐F_1_ (+)	19.2 (1.0)	**17.2 (0.6)** a	**55.0 (3.1)** a	25.0 (0.2)
WH2‐F_1_ (−)	19.1 (1.2)	13.4 (0.9)	44.1 (2.7)	24.1 (0.4)
WH3‐F_1_ (+)	14.3 (0.9)	12.4 (0.6)	48.6 (3.5)	22.0 (0.6)
WH3‐F_1_ (−)	15.4 (1.0)	10.9 (0.7)	38.8 (5.4)	22.1 (0.7)
WH4‐F_1_ (+)	23.7 (1.7)	18.6 (1.0)	44.6 (2.0)	23.0 (0.4)
WH4‐F_1_ (−)	23.6 (1.6)	17.7 (0.9)	37.2 (4.4)	22.1 (0.4)
WH5‐F_1_ (+)	18.1 (0.8)	13.9 (0.5)	40.5 (5.9)	23.1 (0.5)
WH5‐F_1_ (−)	17.6 (1.4)	12.5 (0.6)	34.8 (3.1)	21.2 (0.9)
WH1‐F_2_ (+)	12.8 (0.6)	**9.5 (0.3)** a	**59.0 (2.0)** a	23.3 (0.3)
WH1‐F_2_ (−)	12.6 (0.4)	8.1 (0.2)	48.8 (1.9)	22.6 (0.4)
WH2‐F_2_ (+)	**15.5 (0.4)** a	**11.6 (0.4)** a	54.0 (3.0)	22.9 (0.3)
WH2‐F_2_ (−)	13.7 (0.5)	9.6 (0.5)	54.8 (2.0)	23.0 (0.3)
WH3‐F_2_ (+)	12.2 (0.4)	9.6 (0.4)	60.3 (1.8)	22.8 (0.2)
WH3‐F_2_ (−)	12.5 (0.5)	8.4 (0.4)	52.1 (4.2)	22.1 (0.7)
WH4‐F_2_ (+)	15.0 (0.7)	11.7 (0.5)	54.9 (1.6)	22.2 (0.4)
WH4‐F_2_ (v)	14.9 (0.5)	10.6 (0.4)	55.5 (2.8)	22.0 (0.5)
WH5‐F_2_ (+)	11.9 (0.5)	8.7 (0.5)	54.5 (2.3)	22.1 (0.3)
WH5‐F_2_ (−)	12.2 (0.5)	8.2 (0.3)	48.1 (2.8)	21.3 (0.4)

a *P *<* *0.05.

**Table 4:** Paired *t‐*test for fitness‐related traits between transgene‐present (+) and transgene‐absent (−) hybrid lineages in mixed planting. Values in brackets indicate standard errors (SE). Values in **bold** followed by asterisks indicate significant differences between the paired transgene‐present and transgene‐absent lineages

Plant materials	No. of tillers per plant	No. of panicles per plant	Ratio of seed set (%)	1000‐seed weight (g)
WH1‐F_1_ (+)	**15.9 (0.6)** a	12.1 (1.1)	**43.8 (2.79)** a	21.7 (0.4)
WH1‐F_1_ (−)	13.4 (1.2)	11.1 (1.5)	29.6 (2.23)	21.0 (0.5)
WH2‐F_1_ (+)	**19.2 (1.1)** a	16.2 (0.9)	**48.8 (2.42)** a	24.9 (0.3)
WH2‐F_1_ (−)	14.2 (0.8)	13.6 (1.0)	40.7 (3.28)	24.2 (0.5)
WH3‐F_1_ (+)	**14.7 (0.7)** a	**13.7 (1.1)** a	35.9 (5.54)	21.2 (0.9)
WH3‐F_1_ (−)	10.1 (1.1)	9.4 (0.6)	32.1 (4.94)	21.0 (0.3)
WH4‐F_1_ (+)	**18.2 (0.9)** a	15.9 (0.9)	43.6 (2.63)	22.3 (0.5)
WH4‐F_1_ (−)	14.2 (1.0)	14.5 (0.9)	37.1 (3.00)	21.2 (0.6)
WH5‐F_1_ (+)	**14.8 (1.2)** a	13.4 (1.4)	37.3 (4.56)	**22.3 (0.3)** a
WH5‐F_1_ (−)	11.5 (0.9)	10.3 (0.6)	31.3 (3.08)	21.2 (0.6)
WH1‐F_2_ (+)	**12.4 (0.7)** a	**9.3 (0.8)** a	61.1 (3.2)	23.6 (0.4)
WH1‐F_2_ (−)	11.5 (0.9)	7.4 (0.6)	56.2 (2.2)	22.8 (0.4)
WH2‐F_2_ (+)	16.0 (1.2)	12.2 (1.1)	60.7 (2.0)	23.0 (0.3)
WH2‐F_2_ (−)	15.6 (0.9)	11.4 (0.9)	56.0 (1.4)	22.7 (0.4)
WH3‐F_2_ (+)	11.7 (0.6)	8.7 (0.5)	57.3 (1.0)	22.9 (0.2)
WH3‐F_2_ (−)	12.3 (0.2)	8.8 (0.2)	54.0 (2.1)	22.0 (0.5)
WH4‐F_2_ (+)	15.4 (0.9)	11.5 (0.8)	56.6 (2.4)	20.7 (0.4)
WH4‐F_2_ (−)	14.5 (0.8)	9.8 (0.7)	53.7 (1.8)	21.0 (0.4)
WH5‐F_2_ (+)	**14.4 (0.99)** a	10.0 (1.0)	51.5 (1.0)	22.2 (0.3)
WH5‐F_2_ (−)	10.8 (0.90)	7.2 (0.6)	49.6 (3.2)	21.2 (0.6)

a *P *<* *0.05.

### The correlation between the observed and simulated fecundity increases in transgene‐present F_1_–F_2_ hybrid lineages

**Figure 4:**
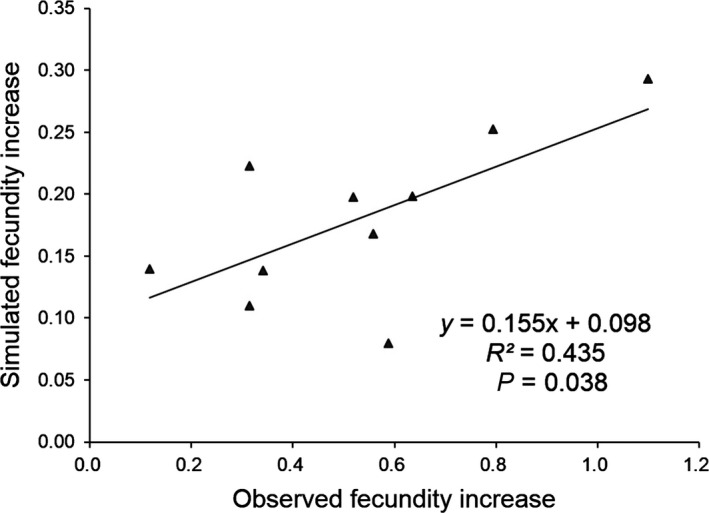
Correlation between the observed fecundity increase and simulated fecundity increase. The observed fecundity increase was recorded in transgene‐present weed‐crop hybrid lineages compared with transgene‐absent lineages. The simulated fecundity increase was calculated as the fecundity response from every 1% insect damage index reduction in weedy rice parents multiplied by the insect damage index from transgene‐absent F_1_ and F_2_ hybrid lineages.

The fecundity response, defined as the reduced percentage of seed production by every 1% increase in insect damage index, varied among the five weedy rice populations (W1–W5), ranging from 0.85% to 2.30% (Table S3). The simulated fecundity advantages in the transgene‐present F_1_ and F_2_ hybrid lineages ranged from 7.9% to 29.3% (Table S3). The observed fecundity increase, which was associated with the introduction of insect‐resistant transgenes in the transgene‐present F_1_–F_2_ hybrid lineages, was not significantly correlated with the insect damage indices in the corresponding transgene‐absent hybrid lineages or the fecundity responses of weedy rice to the target insects. However, the observed fecundity increase was significantly correlated with the simulated fecundity increase in the transgene‐present F_1_ and F_2_ hybrid lineages (Fig. 4). Additionally, the weighted fecundity increases among these lineages were positively correlated (Pearson coefficient 0.645, *P* < 0.05) with the fecundity responses of weedy rice to the target insects.

## Discussion

### Insect‐resistant transgene increases fecundity in crop‐weed hybrid lineages under high‐insect pressure

A relatively high level of insect pressure in the high‐insect environment and a low level of insect of insect pressure in the low‐insect environment were established in this study, based on the insect damage indices. These results justify a comparison of the fitness differences associated with insect‐resistant transgenes in the rice crop‐weed hybrid lineages. We preferentially focused our estimation on the fitness benefit of the insect‐resistant transgenes because of concerns about increased weedy rice infestation resulting from transgene flow (Cao et al. 2009; Lu and Yang 2009; Yang et al. 2011, 2012, 2015; Wang et al. 2014).

The three‐way anova evidently indicated a significant effect (*P *<* *0.001) of the insect‐resistant transgenes (*Bt/CpTI*) on the performance of fitness‐related traits in the crop‐weed hybrid lineages. As a result, the damage by target insects was significantly reduced in the transgene‐present hybrid lineages, resulting in considerably increased fecundity under high‐insect pressure, compared with the weedy parents and transgene‐absent hybrid lineages. These results are in general agreement with previous studies on transgenic sunflowers (Snow et al. 2003) and weedy rice (Yang et al. 2011, 2012, 2015), where enhanced fecundity associated with the insect‐resistant transgenes was observed under high or natural insect pressure, although in our case, considerable variation of the benefits was observed among hybrid lineages and different years. The increased fitness benefit for weedy rice populations by the introduction of insect‐resistant transgenes may provide the weedy populations with opportunities for evolution, provided that the insect pressure in the ambient environment is chronically high. Such evolution may translate into increased weed problems or other undesirable ecological impacts (Lu and Snow 2005; Lu and Yang 2009; Ellstrand et al. 2013). Therefore, effective methods for mitigating the fitness advantages of the insect‐resistant transgenes in weedy/wild rice populations should be considered to reduce such an undesired impact (Gressel and Levy 2014; Gressel 2015).

### Ambient insect pressure and genotypes of weedy rice parents determines the fecundity change from transgenes

In this study, insect‐resistant transgenes (*Bt/CpTI*) significantly reduced target insect damage, increasing the fecundity of transgenic crop‐weed hybrid progeny under high‐insect pressure. Our results are supported by previous studies on the risk assessment of transgene flow from GE crops to their wild relatives (Snow et al. 2003; Vacher et al. 2004; Halfhill et al. 2005; Yang et al. 2011). Also, we found ambient insect pressure itself influenced the fecundity of transgene‐present insect‐resistant crop‐weed hybrid progeny; we observed lower target‐insect pressure in the mixed‐planting plots than in the pure‐planting plots, with an average of ~5% reduction in both the F_1_ and F_2_ hybrid lineages. As a consequence, the observed fecundity increase in the transgene‐present hybrid lineages was less pronounced in the mixed‐planting plots than in the pure‐planting plots. This trend is most likely due to the reduced ambient insect pressure caused by the mixed insect‐resistant plants containing transgene; as reported in previous field studies, a considerably reduced level of target insects was observed in the mixed‐planting plots with transgene‐present plants (Yang et al. 2011, 2012, 2015). Wu et al. (2008) also reported the generally reduced insect populations in the commercial production fields where insect‐resistant *Bt* cotton varieties were cultivated for more than ten years. We therefore predict that even though the insect‐resistant transgenes (*Bt*,* CpTI*) will be incorporated into weedy rice through gene flow in the commercial GE rice fields, its fitness advantage is neglectable because the target insect pressure will be inhibited by the extensively cultivated transgenic plants.

Notably, our three‐way anova indicated a significant effect of weedy rice genotypes on the fitness‐related traits. The considerable variation in fitness of the crop‐weed hybrid lineages with different parents also suggests the important roles of genotype of the transgene‐flow recipients in responding to insect attacks and consequently affecting fitness differences. As suggested by Selvi et al. (2002), weedy or wild recipients of different genotypes may have evolved diverse tolerances to the target insects, which can considerably affect the damage of the weedy or wild plants under the same insect pressure. If a weedy rice parent has the genetic background that is originally more resistant to target insects, the insect‐resistant transgenes may convey a lower fecundity advantage to its crop‐weed hybrid progeny. It is true that in this study as the weighted fecundity increases among hybrid lineages are positively correlated with the fecundity responses to target insects of the weedy rice. Based on our findings in this study, we can explain the reason for the contradictory results of fitness effect caused by transgene flow in previous studies (e.g., Snow et al. 1999, 2003; Guéritaine et al. 2002; Burke and Rieseberg 2003; Halfhill et al. 2005). We would point out that the insect pressure (environment) and the fecundity responses to target insect by weedy rice recipients (genotypes) work together to determine the fecundity change, as revealed by the significant correlation between the observed fecundity increase and the simulated fecundity increase. Consequently, significant differences in fitness‐related traits between transgene‐present and transgene‐absent hybrid lineages were detected in plants with low‐insect resistance and/or with high‐insect damage indices. To our knowledge, this is the first study specifically addressing the significance of genetic background on fitness variation in crop‐weed hybrid progeny containing insect‐resistant transgenes. This finding has profound implications in the biosafety assessment for transgene flow. Given the presence of great genetic diversity in weedy/wild rice populations (Cao et al. 2006; Londo and Schaal 2007; Vaughan et al. 2008; Shivrain et al. 2010; Xia et al. 2011a), as well as in wild relatives of other crops (e.g., Cronn et al. 1997; Sasanuma et al. 2002), ecological consequences caused by the movement of the same transgene to various weedy/wild populations may considerably different. Thus, it is not appropriate to draw a general conclusion concerning the environmental impact from transgene flow based on field experiments involving only a single genotype of a transgene recipient.

In conclusion, our present field experimental study indicates that the insect‐resistant transgenes (*Bt/CpTI*) can significantly increase the fecundity of transgene‐present crop‐weed hybrid lineages in the environment with relatively high‐insect pressure. This finding agrees with the results of previous studies (Yang et al. 2012, 2015). However, the fecundity increase showed considerable variation among hybrid lineages derived from different weedy rice parents, indicating the key role of the genotypes of transgene‐flow recipients in affecting fitness advantages. Field experiments in this study also indicate the key role of ambient insect pressure that can considerably influence the fitness results of the transgene‐present crop‐weed hybrid lineages. Obviously, factors such as the environment to which the transgenic plants expose, genotypes of the transgene flow recipients, and their interaction will considerably influence the field performance of crop‐wild/crop‐weed hybrid progeny containing insect‐resistant transgenes for fitness evaluation. Therefore, it is inappropriate to draw a general conclusion for the risk assessment of transgene flow to crops’ weedy/wild relatives through fitness evaluation based on a single experiment that only involves a limited number of transgene flow recipients as the weedy/wild parent under a particular environmental condition.

## Supporting information


**Table S1.** Weedy rice populations and cultivated rice lines used for producing crop‐weed hybrids with information on their country of origin, seed shattering trait, and growth duration.
**Table S2.** Methods for measuring the selected fitness‐related traits and the insect damage index in this study.
**Table S3.** Fecundity responses of weedy rice parents to insect damage index in transgene‐present (+) and transgene‐absent (−) plants, simulated fecundity increase, and the observed fecundity increase of F_1_ and F_2_ hybrid lineages.
