# Supplementary material for: Ambient insect pressure and recipient genotypes determine fecundity of transgenic crop‐weed rice hybrid progeny: Implications for environmental biosafety assessment
Source: Evol Appl. 2016 Mar 2;9(7):847–56. doi: 10.1111/eva.12369 (PMC4947147; doi:10.1111/eva.12369)
Supplement: Supplementary file 1 — Table S1. Weedy rice populations and cultivated rice lines used for producing crop‐weed hybrids with information on their country of origin, seed shattering trait, and growth duration. Table S2. Methods for measuring the selected fitness‐related traits and the insect damage index in this study. Table S3. Fecundity responses of weedy rice parents to insect damage index in transgene‐present (+) and transgene‐absent (−) plants, simulated fecundity increase, and the observed fecundity increase of F1 and F2 hybrid lineages. [file EVA-9-847-s001.doc]

**Table S1.** Weedy rice populations and cultivated rice lines used for producing crop-weed hybrids with information on their country of origin, seed shattering trait, and growth duration.

| Plant material | Code | Country of origin | Seed shattering ability*^1^* | Approximate duration (day)*^2^* |
| --- | --- | --- | --- | --- |
| Weedy rice population 1 | W1 | Nepal | Low | 120 |
| Weedy rice population 2 | W2 | Vietnam | High | 102 |
| Weedy rice population 3 | W3 | China | Low | 98 |
| Weedy rice population 4 | W4 | South Korea | Moderate | 95 |
| Weedy rice population 5 | W5 | India | Moderate | 98 |
| Insect resistant rice with *Bt/CpTI* transgenes (Kefeng8MF63) | MF1 | China | Persistent | 101 |
| Non-transgenic rice (parental line of MF1) | M86 | China | Persistent | 100 |

*^1^* Persistent: seed shattering < 5%; Low: seed shattering < 40%; Moderate: seed shattering between 41-80%; High: seed shattering >80%.

*^2^* Days from seed germination to the first flowering in the early rice season measured in Fuzhou, Fujian Province, China.

**Table S2. Methods for measuring the selected fitness-related traits and the insect damage index in this study.**

| Trait | Methodology |
| --- | --- |
| Number of tillers per plant | Measured 60 days after transplanting, blasted tillers were included |
| Number of panicles per plant | Measured when plants were matured, white heart or late panicles were excluded |
| Number of good seeds per plant | Count the number of well filled seeds after harvest |
| Seed set rate (%) | Calculated as: Number of good seeds/ Number of total spikelets *100% |
| 1000-seed weight | The weight of 1000 seeds, seeds were dried for 5 days in 55℃ |
| Folded leaf rate (%) | Calculated as: Number of folded leaves/total number of leaves*100% |
| Blasted tiller rate (%) | Calculated as: Number of blasted tillers/total number of tillers*100% |
| Insect index (%) | Calculated as: (folded leaf rate + blasted tiller rate)/2 |

**Table S3.** Fecundity responses of weedy rice parents to insect damage index in transgene-present (+) and transgene-absent (−) plants, simulated fecundity increase, and the observed fecundity increase of F_1_ and F_2_ hybrid lineages. The simulated fecundity increase was calculated as: [insect damage index in transgene-present plants – insect damage index in transgene-absent plants] × fecundity response of weedy rice. The weighted fecundity increase was calculated as [observed fecundity increase/ insect damage index].

| Crop-weedy combination | Fecundity response of weedy rice (%) | Insect damage index in transgene-present plants | Insect damage index in transgene-absent plants | Simulated fecundity response | Observed fecundity increase | Weighted fecundity increase |
| --- | --- | --- | --- | --- | --- | --- |
| WH1-F_1_ | 2.30 | 0.56% | 13.30% | 0.293 | 1.099 | 8.6% |
| WH2-F_1_ | 0.88 | 0.39% | 22.85% | 0.198 | 0.635 | 2.8% |
| WH3-F_1_ | 1.23 | 1.41% | 15.10% | 0.168 | 0.558 | 4.1% |
| WH4-F_1_ | 1.97 | 2.90% | 14.22% | 0.222 | 0.314 | 2.8% |
| WH5-F_2_ | 0.85 | 1.89% | 18.24% | 0.139 | 0.117 | 0.7% |
| WH1-F_2_ | 2.30 | 4.60% | 15.6% | 0.252 | 0.794 | 7.2% |
| WH2-F_2_ | 0.88 | 4.29% | 16.7% | 0.109 | 0.314 | 2.5% |
| WH3-F_2_ | 1.23 | 3.12% | 19.2% | 0.198 | 0.519 | 3.2% |
| WH4-F_2_ | 1.97 | 3.47% | 10.5% | 0.138 | 0.341 | 4.9% |
| WH5-F_2_ | 0.85 | 4.55% | 13.9% | 0.079 | 0.588 | 6.3% |
